# Water palatability, a matter of taste

**DOI:** 10.1186/s40813-015-0004-z

**Published:** 2015-07-29

**Authors:** Manon A. M. Houben, Arie van Nes, Tijs J. Tobias

**Affiliations:** 1 PorQ BV, P.O. Box 52, 5690 AB Son, The Netherlands; 2 Department of Farm Animal Health, Utrecht University, Faculty of Veterinary Medicine, Yalelaan 7, 3584 CL Utrecht, The Netherlands

**Keywords:** Swine, Water, Taste, Additives, Organic acids, Latin Square design

## Abstract

**Background:**

The aim of this trial was to test whether the temperature or additives of the drinking water affected water uptake by nursery pigs. We designed a repeated 4 × 4 Latin Square to control for confounding factors such as; carry-over effects, learning of a preferential taste, daily variation within groups and regular increase of uptake over a day due to diurnal drinking patterns. Water types tested were control water (A); warm water (33 °C); (B); organic acid additive 1 (C), and organic acid additive 2 (D).

**Results:**

The piglets drank more of water C than of control water (A). The uptake of water D was marginally higher than control water (A). There was no difference in uptake of water B and A. However, a learning effect was observed resulting in increasing amounts of water type C and D taken up over the four consecutive days. A carry-over was not fully prevented as pigs always consumed less during the second hour and water D was consumed less during the fourth and final hourly observation period each day.

**Conclusions:**

The experimental design can be used in future trials for evaluation of the water uptake and preference of water additives for pigs. The tested commercial organic acid additives did not adversely affect water uptake of drinking water, water uptake increased instead.

**Electronic supplementary material:**

The online version of this article (doi:10.1186/s40813-015-0004-z) contains supplementary material, which is available to authorized users.

## Background

Sufficient water uptake is essential for pig health and production. Water uptake is affected by physiological, biochemical, nutritional and behavioural requirements, as well as by drinking water quality [1]. Water quality is determined by the specific constituents as well as by the drinking water system that also affects palatability. For instance, a low pressure water system installed in a warm environment (e.g. barn for weaner pigs) may cause an increase in drinking water temperature and may therefore affect water uptake.

Whereas it is known which water constituents may have an adverse effect on water palatability or health [2], it is, however, not well known what the effects of pharmaceutical or additive products are on water acceptance and or water uptake, because pigs are regularly not given a choice of water to drink. Maybe, if water palatability and uptake can be improved, this may also improve production, as there is a correlation in uptake of feed, mostly by crude protein, and water [3]. Water taste preference studies have been performed with natural and artificial sweeteners [4]. However, the taste preferences of pigs for acid additives to drinking water is unknown. Also is unknown whether the temperature of drinking water has an effect on the water uptake. Extrapolating water preferences from man to pigs seems invalid, as pigs differ from man in the number and distribution of taste receptors [5], which may result in different taste preferences [4]. A preference of water temperature may be associated with ambient temperature [6] and production level [7].

Water additives, for instance containing organic acids that lower pH, are often used for control of enteric pathogens [8]. Whether pH of water affects palatability and thus uptake is uncertain as reports on this matter are contradictory. Some reported in comparison with plain water no reduction of uptake of drinking water with added lactate and pH of 3.2 [9, 10]), whereas de Busser et al., reported a significant decrease in water uptake at a pH of 4 compared to pH of 8 [8] when using a mixture of organic acids. Therefore, it is unclear whether the water uptake is affected by solely pH or by specific product constituents affecting osmolality, palatability or odour.

A classical preferential taste experiment usually comprehends observations on individual pig’s choices, and may be difficult to extrapolate to field situations. Alternatively, water uptake as a parameter for water preference is much more feasible to study under field conditions. If we keep in mind that many possible confounders can interact with water uptake as well: body weight [11], disease, feed constituents (mainly protein [12, 13], sodium and potassium [10, 14, 15]) and ventilation (e.g. due to evaporation by respiration [11]), ambient [6, 11] and water temperature [6]. The method of distributing drinking water can affect water disappearance as well, by type [16], the number of nipple drinkers per number of pigs [17] and, likely due to neophobia, also the shape and colour of the drinker [18]. Next, the uptake of water follows a diurnal pattern [17–19] with increasing water uptake in the evening. Finally, pigs may express behaviour to consume additional water beyond the physiological needs [20].

The objective of this study was (i) to design an experimental setup that may detect a difference in water uptake under farm conditions while accounting for known confounders and (ii) to evaluate the preference of weaned pigs for water with two different water temperatures and water with two commercially available water additives. To study the uptake of four types of water (A: control water; tap water of 10 °C, B: warmed tap water (approximately 33 °C), C: tap water with 0.1 % concentrate of commercial organic acid product nr. I, D: tap water with 0.1 % concentrate of commercial organic acid and etheric oils product nr. II. a Latin Square experimental design was used and water uptake was measured while controlling for the aforementioned confounding factors.

## Results

### Water temperature and pH

Water pH varied slightly per observation (Table 1), especially pH of water types A and B due to low buffering capacity of the water. With respect to water temperature, it either increased (water types A, C and D) or decreased (type B) during the hour due to the ambient temperature (of 23 °C) in the pig barn (Table 1).

**Table 1:** pH en temperature of water types

Water type	pH average	pH range	Temperature start average	Temperature start range	Temperature after 1 h average	Temperature after 1 h range
A	7.3	7.0 ; 7.8	10.7	9.0 ; 15.0	16.2	15.4 ; 17.0
B	7.2	6.7 ; 7.8	33.4	31.9 ; 35.7	26.2	24.0 ; 28.5
C	3.7	3.5 ; 3.8	10.4	9.0 ; 14.4	16.2	15.4 ; 17.0
D	3.6	3.4 ; 3.7	10.4	9.0 ; 14.0	16.3	15.8 ; 17.1

### Water uptake

The average water uptake per pig per hour was 0.21 kg/pig/h (inter quartile range 0.16–0.26) (See Additional file 1). Excessive spillage or play behaviour with water in the drinkers was not observed. Analysis of Variance showed no significant difference in water uptake between pens (Table 2), or between pig sex (not shown). Significant effects of day, time of day and water type on the water uptake were observed using ANOVA and all were retained in the multivariate regression model (Table 3).

**Table 2:** Result of ANOVA analysis

Bron	Df	Sum of squares	Mean sum of sq	F-value
Total	63	0.365	0.005794	
Water type	3	0.146582	0.048861	17.84^***^
Day	3	0.033395	0.011132	4.06^*^
Time of day	3	0.042320	0.014107	5.15^**^
Pen	3	0.003003	0.001001	0.36
Residuals	51	0.139705	0.002739	NA

^*^ *P* < 0.05; ^**^ *P* < 0.01; ^***^ *P* < 0.001

**Table 3:** Results of multivariate linear regression model for water uptake.

	Estimate	Std. Error	T-value	*P*-value	99 % CI low	99 % CI high
Intercept	0.182	0.024	7.694	<0.001	0.120	0.242
Water type B	−0.005	0.019	−0.252	0.802	−0.052	0.043
Water type C	0.115	0.019	6.194	<0.001	0.067	0.162
Water type D	0.043	0.019	2.338	0.023	−0.004	0.091
Day 2	−0.000	0.019	−0.022	0.982	−0.048	0.047
Day 3	0.015	0.019	0.831	0.410	−0.032	0.063
Day 4	0.056	0.019	3.008	0.004	0.008	0.103
Time of day 2	−0.068	0.019	−3.650	<0.001	−0.115	−0.020
Time of day 3	−0.047	0.019	−2.567	0.013	−0.095	0.000
Time of day 4	−0.021	0.019	−1.109	0.273	−0.068	0.027
Pen 2	0.010	0.019	0.527	0.600	−0.038	0.057
Pen 3	0.019	0.019	1.009	0.318	−0.029	0.066
Pen 4	0.014	0.019	0.739	0.463	−0.034	0.061

The intercept is the estimate for day 1, time of day 1 and water type A in kg water uptake per pig per hour. In the latter two columns the 99 % confidence interval estimates are given

**Fig. 1:**
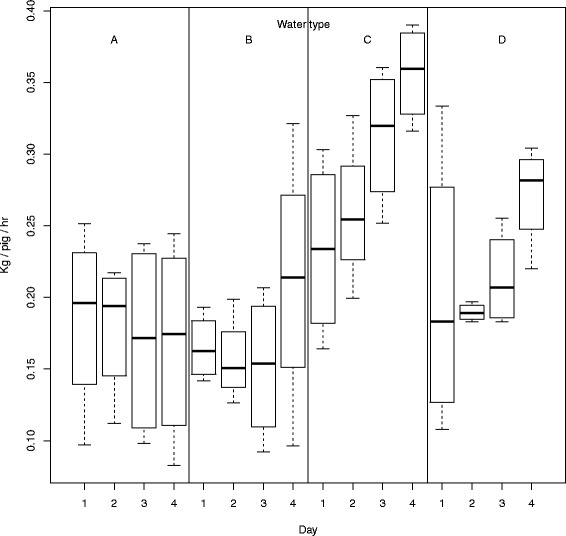
The uptake of water type per pig per hour at each day

**Fig. 2:**
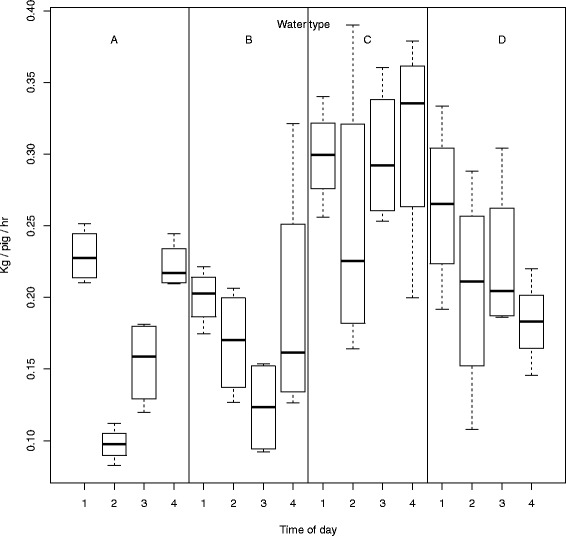
The uptake of water type per pig per hour during different times of the day

Day: on the fourth day significantly more water was consumed than on day 1, 2 or 3 (Fig. 1 and Table 3). Time of day: during the first hour of each day significantly more water was consumed compared to the second and third hour, but not compared to the last hour of the day (Fig. 2 and Table 3). Water type: water uptake of water C (*P* < 0.001) and D (*P* < 0.05) were significantly higher than uptake of control water (Figs. 1 and 2 and Table 3). If correction for multiple comparisons is applied, the P-value for uptake of water type D may be multiplied with three and thus *P* = 0.07. Consumption of warm water (B) was not different from control water.

The uptake of the different water types seemed to be affected by day and time of day. Water uptake of water A and B was more or less equal on each day, whereas water uptake of water C and D increased each day (Fig. 2). Over the day, water uptake differed between water types as well (Table 3). Consumption of water C was on a relative high level all times, irrespective of time of day. However, other water types showed to result in lower uptake in the second hour compared to the first hour. Stated differently, water uptake was higher for each water type in the first hour than in the other hours, except for water C. Water D always resulted in higher uptake than water A, except in the fourth hour (Fig. 2 and Table 3).

Whereas pen did not significantly affect water uptake, it seemed that water B was consumed slightly better in pen 3 and 4 than in pen 1 and 2 (results not shown). The uptake of water C and D was higher in each pen, but the uptake of water D seemed to be slightly lower than that of water C.

## Discussion

In this study we found that the organic acid containing additives did not adversely affect water uptake in seven week old pigs and that there seems a difference in preference for such products. The results showed a significant increase in water uptake of water type C (organic acids), whereas the uptake of warm water was not different from control water. Whether the uptake of water D (organic acids with etheric oils) is significantly higher can be discussed. Furthermore, we conclude that the latin square design is a valid method to test the uptake and preference of different water constitutions under field conditions. Whereas it was aimed to account for confounding factors, there is some room for improvement in the design for future trials.

The results showed a difference in water uptake between water types, but the day and time of day also explained some of the variation, despite that we aimed to control for this variation by the experimental design. It was assumed that between water types there would be no ‘carry-over’ effect, that there would be no learning effect over the four days, and that by repeating the experiment over multiple days the effects for time or day would be controlled for. However, the results suggested that there was a carry-over effect. This may be explained in two ways. Firstly, in the second hour of the day water uptake was significantly reduced which is contradicting with an increase in uptake in the diurnal uptake scheme. The reduced uptake during the second hour may be caused by an excess of uptake during the first hour as a result of curiosity or play behaviour towards a new stock of fresh water or due to researchers’ activities before commencement of the trials daily. During the experiment much more activity was indeed observed around the bowls during the first hour than during consecutive times of day. Secondly, the uptake of water D in the fourth hour was always less than on other hours of the day. If water C was indeed more preferred by the pigs, likely pigs may already had taken up more than enough water earlier that day resulting in some kind of satiated effect and lower uptake of water D. When one would like to control for the carry-over effect by statistical methods, the suggested experimental setup should be expanded, with more pens for instance, to have more data to be able to include more explanatory variables.

The results also suggest that for some acidified waters a learning period is needed. Water uptake of water C and D increased day by day, whereas this was not the case for water A or B. While very subjectively, it seemed that during the experiment pigs were more anxious when provided with water C or D later in the week. Moreover, uptake of water B was much lower at the beginning of the week, but in the end there was no significant difference. Either pigs got used to consume warm water. Or, as suddenly there was one very cold night on the third day to the fourth day, an increase in uptake of warm water has diminished a possible negative effect of warm water in the statistics. This would corroborate with reports that pigs can use water for temperature homeostasis as well [10]. If there is a learning effect, this may actually justify the experimental design, as a single observation per group would not have detected this and using observations on total uptake of water over a longer period may also not have detected this effect.

With this experimental design it seems possible to compare the pig’s preference of different water constitutions under field conditions, but recommendations for future trials are:

- To start daily with an extra round of ‘control’ water to create a feeling of satiation in the pigs and prevent confounding by explorative behaviour.
- Consider a ‘washing’ step with control water between water types to reduce the carry-over effect. Whereas a somewhat longer period between subsequent water types may be desired to reduce a carry-over effect, this will increase the total daily observational period and thus may also induce more variation because of the diurnal drinking pattern. Also there could be an effect on intake due to play behaviour and curiosity which may be induced for each observational period on that day.
- Ideally, the test is performed somewhat later on the day to obtain higher absolute values; according to Madsen between 15:00 h en 20:00 h [19]. When one chooses to increase a washing step and start observations during the first short and small peak in uptake in the morning [19], one has to analyse the farm specific uptake pattern before the trial to ensure that the observational period does not include a period of reduced intake.

In this experiment it was shown that water C was taken up significantly more than control water and the uptake of water D was marginally different. We conclude that in this trial pigs preferred water C most. However, as water uptake could also be influenced by feed and water composition, translation to general pig population should be performed with caution. In this trial we used both products up to the instructions of the manufacturer, which aim to lower pH of water below 4. Both products contain dissolved salts of organic acids. As salt content acts on the thirst perception, differences in salt contents may cause a difference in uptake. Alternatively, a comparison could have been bade based on the products containing equal salt contents in the water. However this would likely have resulted in different pH and a different in tastes, as salt is also used as a flavour enhancer. We chose to compare the products based on the manufacturer’s instructions and thereby more or less equal pH as this is what will likely happen in the field. Manufacturers of water additives may consider using both approaches before bringing a product to the market. Whereas it is concluded that water C was best preferred by the pigs this does not imply that water C will improve welfare and production, but nor will water D. Studies with long lasting exposure will have to conclude on those issues as well.

## Conclusions

This study presents an experimental design with additional recommendations to evaluate preference of water additives under farm conditions. Water with a commercially available additive of organic acids was preferred over control water by the pigs in this trial.

## Methods

### Setting

The experiment was conducted at the Academic Facility Farm Animal Health “The Tolakker” of Utrecht University, a conventional 190 sow multiplier farm. One compartment for weaned pigs, containing 4 pens of 20 pigs each. On this farm weaned pigs are fed a commercial creep feed ad libitum, which was continued during the trial. During the trial there was no transition of feed.

At weaning, piglets were randomly allocated to pens, stratified by sex, resulting in two pens with boars and two pens with gilts. To prevent interference between the weaning process and water disappearance, the trial commenced when pigs were approximately 47 days old, three weeks after weaning. After weaning, but before commencement of the experiment, in one pen one pig died due to meningitis and another one was euthanized because of recurrent arthritis. Thus, in pen 1 only 18 pigs were present. The indoor temperature during the trial was more or less constant at 23 °C.

**Fig. 3:**
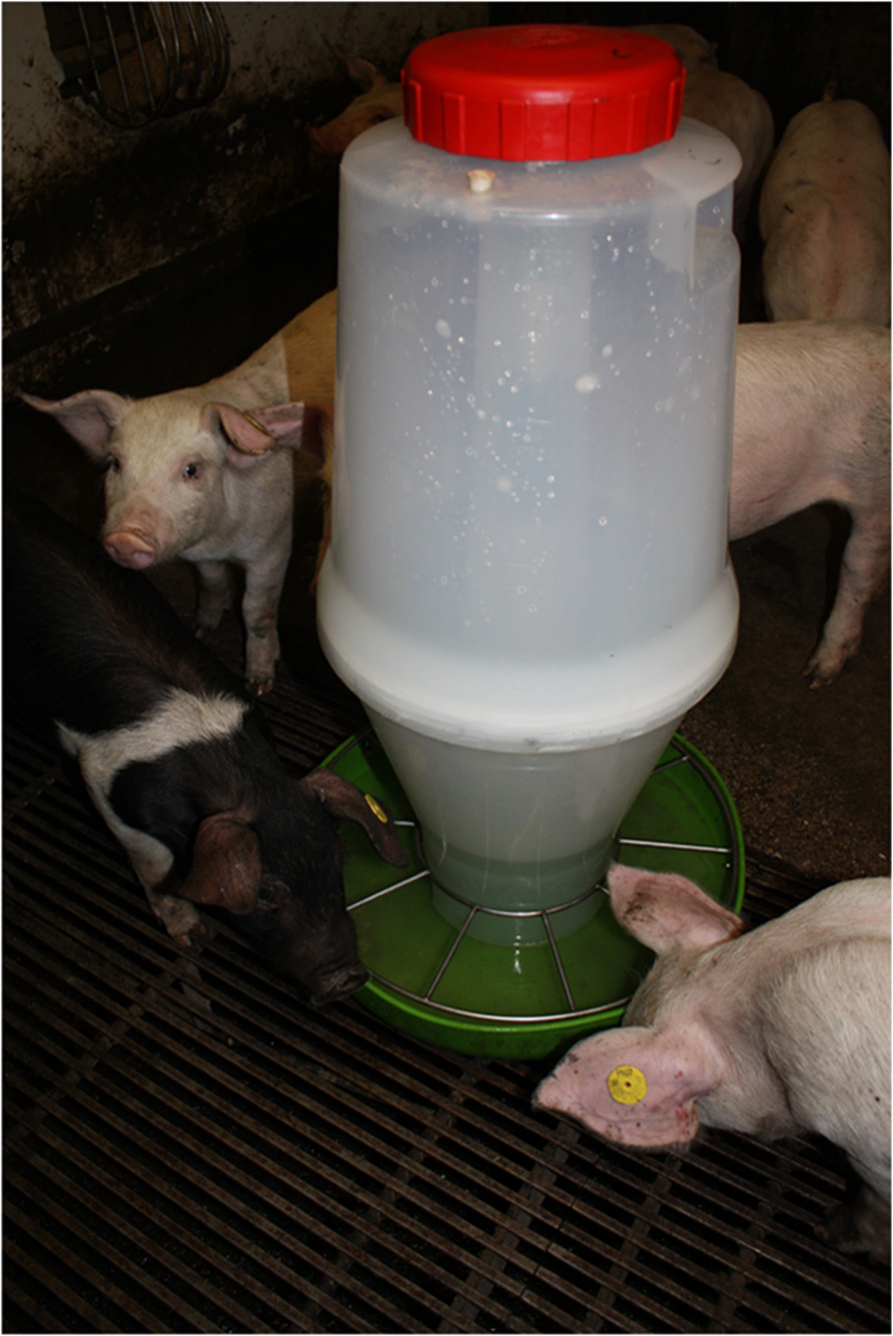
Photo of the bowl drinker with storage container, used in the experiment

On this farm, water is obtained from the municipal drinking water system, with a small storage tank located centrally on the farm. Normally pigs drink water from two low pressure nipple drinkers per pen. However, each day fifteen minutes before the start of the experiment, water supply from the drinking nipples was stopped. For the experiment pigs had to drink from a round water bowl with storage container (Fig. 3). Four days before commencement of the trial the water bowl was placed in the pen as an additional source of water to get pigs used to the drinker and to prevent confounding by exploratory behaviour and spillage of water during the trial. The water bowl and container were cleaned daily before commencing the experiment.

Commonly, an organic acid additive is added to drinking water of weaned pigs on this farm, but for this experiment water treatment was not started until after the experiment had ended to prevent interference with the trial due to acquaintance with the to be tested product.

### Experimental design

Four pens (1 to 4) were given each water type (A–D) each day for one hour, but the order in which the water types were provided, changed daily using a repeated 4 × 4 Latin Square design for four consecutive days (Table 4). By using this setup it was aimed to account for confounding by carry-over, learning of a preferential taste, daily variation within groups and regular increase of uptake over a day due to diurnal drinking patterns [19]. It was assumed that over a four day period confounding due to increase in body weight [11] could be considered negligible. Water was provided in a round bowl (Fig. 3), so confounding by fluctuations in water flow rate and social status at the drinker was prevented. Moreover, spillage is assumed less with bowl drinkers than with nipple drinkers [21] and by using a bowl it was possible to easily measure the amount of water taken up. Water spillage and play behaviour with water was observed qualitatively.

**Table 4:** Schematic overview of order of provision of types of water, using a repeated 4 × 4 Latin Square design

Time start	Day 1				Day 2				Day 3				Day 4			
	Group 1	Group 2	Group 3	Group 4	Group 1	Group 2	Group 3	Group 4	Group 1	Group 2	Group 3	Group 4	Group 1	Group 2	Group 3	Group 4
13:00	B	C	A	D	C	A	D	B	A	D	B	C	D	B	C	A
14:15	D	A	B	C	A	B	C	D	B	C	D	A	C	D	A	B
15:30	A	D	C	B	D	C	B	A	C	B	A	D	B	A	D	C
16:45	C	B	D	A	B	D	A	C	D	A	C	B	A	C	B	D

*A* water type A, control, *B* water type B, warm water 33 °C, *C* water type C, pH 3.7, product I, *D* water type D, pH 3.6, product II

The water types studied were (Table 1): A. Regular cold tap water, B. Warmed tap water, at approximately 33.4 °C, C. Tap water with 0.1 % of commercial additive 1, containing organic acids and a pH of 3.7 and D. Tap water with 0.1 % of commercial additive 2, containing organic acids and etheric oils with pH of 3.6. Commercial additives 1 and 2 were used upon specific instructions of the manufacturer. Both manufacturers had first determined the buffer capacity of the regular tap water three weeks before the experiment to advise on a proper dosing regimen; both recommended to use a concentration of 0.1 % of their product.

The experiment was conducted daily between 13:00 and 18:00 h. At 12:30 h the water containers were removed and cleaned. Water uptake was calculated based on weight of water in the containers. The scale had an accuracy of 5 g. At 13:00 h the containers were placed back in the pen, filled with 10 kg of a particular water type. After exactly 1 hour the container was removed, the remaining water was weighed back and the container was cleaned manually with tap water. Thereafter the container was filled again with 10 kg of the same water type, but placed in another pen exactly 15 min later. Water temperature and pH were measured before placement as well as after removal of the container. Each container was allowed to contain only one water type, to prevent carry-over of taste by residues or odour between water types or containers. As water uptake is associated with ambient temperature, ambient room temperatures were recorded daily before and after executing the experiment. Room temperature was more or less constant during the experiment at 23 °C.

### Statistical analyses

The outcome parameter was the average water disappearance of each type of drinking water per hour per pig in kg. Day, time of day, pen number, sex of pigs in pens and water type were considered as explanatory categorical variables. Using ANOVA the variation of drinking water uptake due to water type, day, time of day, sex and pen was analysed. A multivariate linear regression analysis was performed with pen as fixed variable to correct for repeated measures on pen level and day, time of day and water type as explanatory variables. For statistical analyses R 2.15 software was used [22].

### Ethics

Before execution of the trial, the experimental design was reviewed by the Animal Welfare Officer of Utrecht University. As pigs were not subject to handling or exposure different from regular farming practice the experiment was considered not to be subject to the Animal Experimentation Act and Institutional approval was considered unnecessary.

## Additional file

Additional file 1:
**Data file (data_water_Houben.csv) of water uptake in kg per pig per hour per observation.**
